# Long-term modulation of cardiac activity induced by inhibitory control over emotional memories

**DOI:** 10.1038/s41598-020-71858-2

**Published:** 2020-09-14

**Authors:** Nicolas Legrand, Olivier Etard, Anaïs Vandevelde, Melissa Pierre, Fausto Viader, Patrice Clochon, Franck Doidy, Denis Peschanski, Francis Eustache, Pierre Gagnepain

**Affiliations:** 1 Neuropsychologie et Imagerie de la Mémoire Humaine, Normandie Université, UNICAEN, PSL Research University, EPHE, INSERM, U1077, CHU de Caen, GIP Cyceron, Caen, France; 2 Service d’Explorations Fonctionnelles du Système Nerveux, CHU de Caen, Caen, France; 3 Imagerie et Stratégies Thérapeutiques de la Schizophrénie, Normandie Univ, UNICAEN, ISTS EA 7466, GIP Cyceron, Caen, France; 4 European Center for Sociology and Political Science (CESSP), Université Paris I Panthéon Sorbonne, HESAM Université, EHESS, CNRS, UMR8209, Paris, France

**Keywords:** Cognitive control, Prefrontal cortex, Forgetting, Autonomic nervous system

## Abstract

Efforts to exclude past experiences from conscious awareness can lead to forgetting. Memory suppression is central to affective disorders, but we still do not really know whether emotions, including their physiological causes, are also impacted by this process in normal functioning individuals. In two studies, we measured the after-effects of suppressing negative memories on cardiac response in healthy participants. Results of Study 1 revealed that efficient control of memories was associated with long-term inhibition of the cardiac deceleration that is normally induced by disgusting stimuli. Attempts to suppress sad memories, by contrast, aggravated the cardiac response, an effect that was closely related to the inability to forget this specific material. In Study 2, electroencephalography revealed a reduction in power in the theta (3–8 Hz), alpha (8–12 Hz) and low-beta (13–20 Hz) bands during the suppression of unwanted memories, compared with their voluntary recall. Interestingly, however, the reduction of power in the theta frequency band during memory control was related to a subsequent inhibition of the cardiac response. These results provide a neurophysiological basis for the influence of memory control mechanisms on the cardiac system, opening up new avenues and questions for treating intrusive memories using motivated forgetting.

## Introduction

Intrusive memories often take the form of distressing images and physical reactions that interrupt ongoing mentation^1^. Difficulty controlling these inappropriate emotional responses is a central concern in several psychiatric conditions^2^. Various symptoms of affective disorders, including worrying, the recall of traumatic experiences in PTSD^3^, unwanted thoughts observed in obsessive–compulsive disorder^4^, and the ruminations observed in depression^5^, are representative of such difficulties. Owing to historical assumptions about the independent and pernicious persistence of suppressed memories, it is often assumed that any attempt to exclude distressing images from awareness is harmful^6^. Suppressed memories may backfire, causing psychological symptoms and aggravating emotional responses associated with those memories, which in turn accentuate the perceived distress^7–9^. However, this conclusion largely comes from observations in psychopathology^10–12^. Maladaptive suppression may be the consequence of the inappropriate deployment of the control system which can be compromised in psychiatric disorders, especially in affective disorders^13,14^, and fail to act protectively like an *immune system of the mind*^15^. Interindividual differences in the ability to engage the control system to adequately combat intrusive memories may mean the difference between precipitating or dampening psychopathological symptoms. This idea has recently been supported by a study highlighting the potentially vital role of memory suppression in promoting resilience following a traumatic experience^16^. It remains unknown, however, whether the adequate deployment of control resources during memory suppression might also influence emotional responses.

Emotions are composed of multiple sub-processes distinguishing between their “cognitive” and “bodily/physiological” aspects. In the late nineteenth century, the psychologist and philosopher William James referred to emotions as feelings accompanying bodily changes^17^. Several different studies have since supported and built on this broad framework of embodiment, positing that bodily afferent and autonomic nervous system signals form the basis of emotional construction^18,19^. Some authors, however, have expressed scepticism about the generative role of bodily changes with regard to emotions^20,21^. Critically, these debates have also questioned the nature of the inhibitory control that is required to reduce emotional distress. If emotions are deeply related to bodily changes, emotion regulation may require the direct or indirect downregulation of these states.

The same question applies to the regulation of emotions associated with unwanted memories targeted by suppression processes. The think/no-think task (TNT)^22,23^ is the most commonly used paradigm to assess memory suppression. This task triggers the recollection of unwanted intrusive memories in an experimental setting and allows to examine the after-effects of their suppression. Memory suppression is the capacity to voluntarily stop or cancel conscious awareness directed toward mental images and thoughts associated with memories triggered by a reminder^24^. As a consequence, the unwanted memory may be inhibited, inducing long-term and persistent forgetting of the rejected traces, including both neutral and emotional memories^24,25^. Forgetting mostly affects later conscious recall and declarative components of the suppressed memory traces^24^, including episodic details^26^ and emotional subjective feelings^27^. However, memory traces are stored across multiple and interacting memory systems^28^ including non-declarative or implicit elements^29^. Suppression also impairs implicit expression of the perceptual or conceptual memory contents^30–32^. However, emotions may also associate in memory traces non-declarative components expressed in the autonomic nervous system. Autonomous activity is communicated via projections to the brainstem, which then project to memory brain regions, including the hippocampus and amygdala complex^33^.

The control of both memory intrusions and emotional response in these regions is underpinned by common neurocognitive processes orchestrated by the right prefrontal cortex^34^. Functional magnetic resonance imaging (fMRI) studies have shown that suppressing negative memories involves the inhibitory modulation of areas supporting memory retrieval, such as the hippocampus^25,35^ or visual cortex^30^, as well as subsets of the emotional system, such as the amygdala^25,30,36^. At the electrophysiological level, previous electroencephalography (EEG) studies of the oscillatory dynamics suggest that suppression decreases synchronization of slow oscillatory activity in the theta range^37,38^, a process presumably disrupting memory representations in the medial temporal lobe^39^ and emotional signature in the amygdala^40^. Suppression of memory and emotion originates from a common right prefrontal cortical mechanism that down-regulates the hippocampus and amygdala in parallel^27^. Critically, a similar top-down modulation is also seen during the direct regulation of emotional responses to negative stimuli. Similarly to memory suppression, emotion regulation also activates the right dorsolateral prefrontal cortex^41–44^ to dampen emotional response in the amygdala^45,46^. Memory suppression and emotion regulation show overlapping localization within the right MFG^34,47^ suggesting that memory control is a core component of the regulation of emotional states^27,47^.

However, we still do not know whether memory control influences the embodied and physiological dimension of emotion, and especially cardiac activity, one of the most important manifestations of emotional reactions^48^. According to the *neurovisceral integration model*^49^, which is a prominent conceptualization of the relationship between the central nervous system and heart rate variability, cardiac vagal control is orchestrated via regulation of amygdalar activity by higher-order brain areas in the cortical hierarchy, such as the dorsolateral^50^ and medial^51^ prefrontal areas, which are also engaged during memory control. In this model, the amygdala is a major efferent source of modulation of cardiovascular response via the (parasympathetic) vagal nerve. The *polyvagal theory*^52^ further proposes that efferent vagal connections mediating parasympathetic modulation of heart activity include a phylogenetically more recent ventral branch. This ventral branch, which may be distinctly mammalian, may selectively modulate heart activity, depending on the cognitive context. This consideration of more complex contexts when modulating autonomic activity suggests that higher-level cognitive processes like attention, inhibitory control or memory, also exert an influence on such presumably automatic reactions.

**Figure 1:**
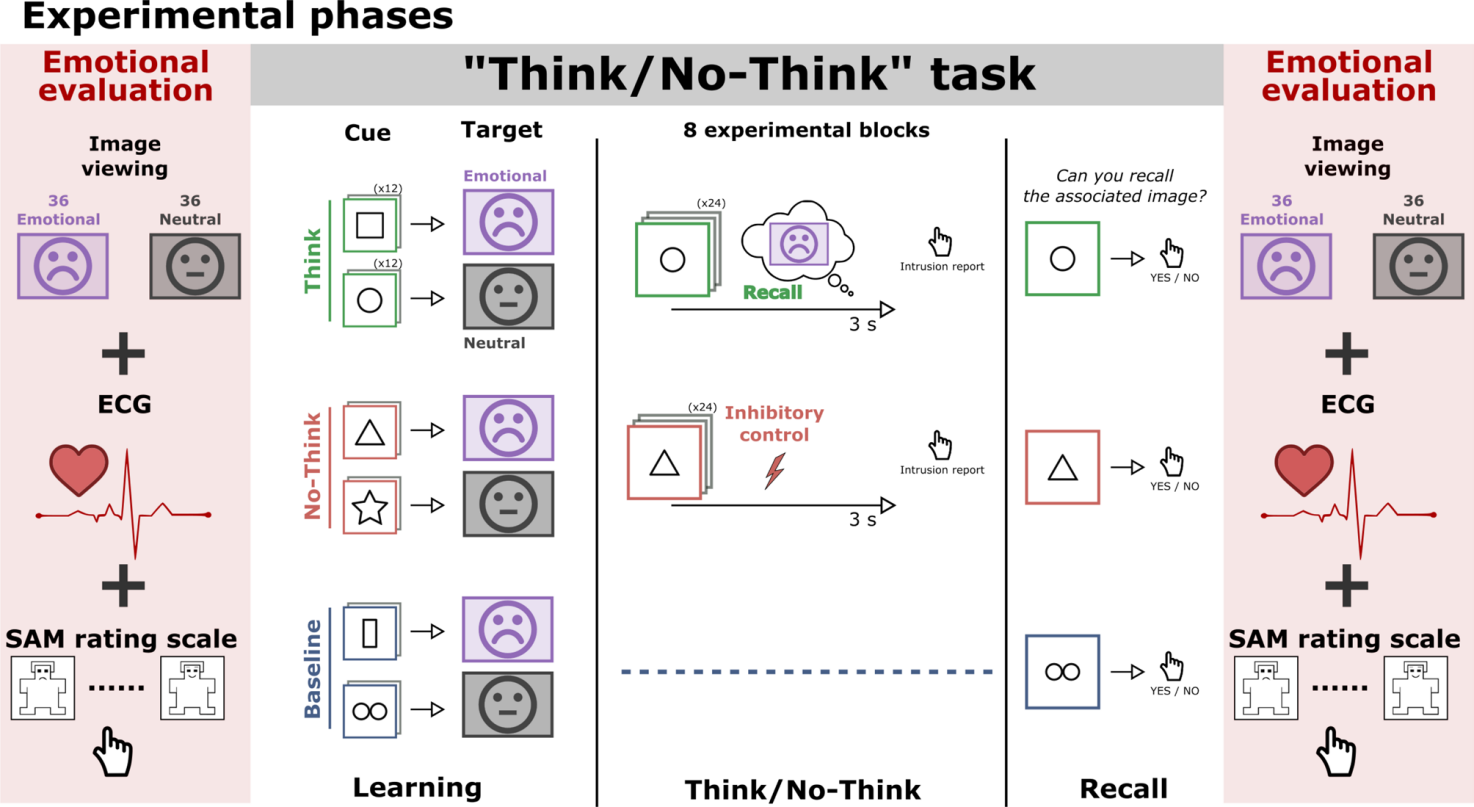
Experimental phases. After learning pairs consisting of pictures of an object (here represented with shapes) and a scene (here represented with neutral and sad faces), participants performed the Think/No-Think (TNT) task. The associated scene had either a neutral or a negative valence. For Think items (in green), participants recalled the associated scene. For No-Think items (in red), they tried to prevent the scene from entering awareness. The baseline items (in blue) were not presented during the TNT task. We recorded subjective valence ratings and cardiac responses to the scenes before and after the TNT task (pre-TNT and post-TNT assessments). A recall task was also administered after the TNT phase, to estimate memory suppression. Supplementary Fig. 1 details the methodological differences between Studies 1 and 2.

Giving these lines of evidence, we postulated that the inhibitory control network engaged to regulate multiple components of the memory traces, including emotion, through the parallel abrogation of hippocampal and amygdalar processes, may also inhibit subsequent cardiac responses to suppressed stimuli. To test this hypothesis, participants underwent electrocardiogram (ECG) measures of their cardiac response to negative and neutral scenes before and after performing the TNT task (Fig. 1) in two consecutive studies. We focused on changes in the intervals between successive heartbeats, referred to as *heart rate variability* (HRV), which are representative of both sympathetic and parasympathetic influences associated with affective responses^53^. Disgust and acute sadness are two prominent primary negative emotions which increase parasympathetic activity or reduce sympathetic influence, inducing cardiac deceleration^54–58^ in individuals experiencing these emotions (see Kreibig et al.^48^ for a review). The origin of cardiac deceleration between these two emotions, however, is likely different. Disgust involves a strong affective response designed to protect us from the risk of disease^59^. Sadness, on the other hand, is associated with complex feelings involving lack of energy, helplessness, or sorrow. Furthermore, disgust enhances episodic memory^60^ and modulates early attentional processes^61^, while sadness does not impact executive functioning or attention^62^. Altogether, these findings suggest that the modulation of memory traces induced by inhibitory control might differ between disgust and sadness and should be examined separately as intended in the current study.

During the TNT task, participants attended to reminders (objects) of these disgusting, sad, and neutral scenes that cued them either to retrieve the scenes (Think items) or suppress their retrieval (No-Think items). During pre- and post-TNT emotional assessments, participants rated the valence of the Think and No-Think scenes, along with previously studied baseline scenes that were not presented during the TNT. During these phases, we tested whether memory suppression was associated with long-term inhibition of the cardiac deceleration that is normally induced by disgusting and sad stimuli. In Study 1, we measured this effect using a block design preserving the separation between Think, No-Think, and Baseline conditions, whereas theses conditions were intermixed in Study 2. A cued-recall task was also performed immediately after the TNT task, to estimate the after-effects of memory suppression on recall performances. In Study 2, participants additionally underwent concurrent electroencephalogram (EEG) measures. The main goal of these EEG recordings was to confirm that the modulation of the cardiac response following memory suppression was truly related to the neural response during the retrieval suppression of memory processes. We focused on theta (3–8 Hz) oscillatory dynamics given their role in suppression. For completeness, we also analysed alpha (8–12 Hz) and low beta (13–20 Hz) ranges which have also been linked to memory suppression^37,38,63^.

## Methods and materials

### Participants

A total of 28 right-handed native French speakers aged 18–35 years took part in Study 1 (14 men), and 24 took part in Study 2 (12 men). They had no reported history of neurological, medical, visual, or memory disorders. The study was approved by the local ethics committee (CPP Nord Ouest I, no. ID RCB: 2015-A01727). All research was performed in accordance with the committee’s guidelines. All participants gave their written informed consent and received financial compensation. Participants were asked not to consume psychostimulants, drugs, alcohol or coffee during the hours preceding the experimental period and to avoid intense physical activity the day before.

### Material

The stimuli were 72 object–scene pairs, plus six filler pairs for TNT practice. The objects were selected from the Bank of Standardized Stimuli^64,65^, and depicted only nonliving and nonorganic artefacts. The scenes were selected from the Nencki Affective Picture System (NAPS) database^66–68^. Half the scenes were categorized as neutral (36 images; mean valence = 5.79, *SD* = 0.84; mean arousal = 4.78; *SD* = 0.71). In Study 1, the other half were categorized as negative, and induced feelings of either disgust (18 images; mean valence = 3.02, *SD* = 0.76; mean arousal = 6.42, *SD* = 0.58) or sadness (18 images; mean valence = 2.96, *SD* = 0.49; mean arousal = 6.43, *SD* = 0.64). In Study 2, the other half all induced disgust (mean valence = 3.15, *SD* = 0.68; mean arousal = 6.35, *SD* arousal = 0.48). Owing to study time limits, we restricted our material to two negative emotions. We selected disgust and sadness, as these dimensions are the most represented in the NAPS database^69^, referring to the discrete quotation provided^67^. As we aimed to obtain a homogeneous set, where each scene depicted as unique a content as possible, we manually added non-categorized images from the NAPS to this selection. For example, some images in the database depict the same scene from different angles. We excluded these pseudoreplicates after visual inspection. The neutral and negative lists were matched on numbers of animals, faces, objects, and people. Each object was paired with a scene, taking care to avoid any pre-existing semantic association (e.g., umbrella as object, and rain in the scene) that might bias the memory encoding. Three lists of 12 pairs corresponding to the Think, No-Think, and baseline conditions were created for each valence condition, and were counterbalanced so that they appeared in each TNT condition equally often, across participants.

### Pre-TNT emotion assessment

To measure their initial emotional reaction to selected scenes and provide a better estimation of the impact of subsequent TNT manipulation on affective responses, participants first performed an emotion rating task. This task consisted in rating both the valence and the arousal associated with each visual scene on the Self-Assessment Manikin (SAM)^70^. This 9-point pictorial scale is labelled with diagram-like manikins with varying emotional facial expressions.

Each scene was displayed against a gray background for a total of 10 s, followed by a 500-ms interstimulus interval. During the first 3 s, the scene was shown on its own, and participants were instructed to carefully explore the scene and understand its meaning. The valence SAM then appeared underneath the picture for 7 s (see Supplementary Fig. 1; the data for the arousal SAM in Study 1 are not shown here). This scale ranges from 1 (deeply unpleasant; frowning face on the far left of the scale) to 9 (pleasant; smiley face on the far right). Using the computer mouse, participants selected the facial expression that most closely matches the scene’s perceived valence. If participants felt neutral (neither happy nor sad nor disgusted), they were instructed to click on the square under the figure in the middle.

We recorded participants’ ECG throughout the 20-min task, using the Biopac MP36R Data Acquisition system and its PC software AcqKnowledge 4.1. For the purposes of another study, participants’ breathing rate and electrodermal activity were also recorded. All the participants undertook this emotional rating task in the morning (8 a.m.) and were familiarized with the task settings using 10 independent images selected from the NAPS database. After an initial resting block of 2 min, the 72 images used in the TNT task (including baseline items) were presented in two blocks of neutral and negative scenes, whose order was counterbalanced across participants. These two main blocks were separated by a second 2-min resting block, to prevent potential overlap and contamination between the cardiac responses to negative and neutral scenes. In Study 1, each block of neutral and negative scenes was further divided into three sub-blocks of 12 images (10 s per image, 126 s per sub-block) corresponding to the Think, No-Think, and baseline conditions. Their order was counterbalanced across participants. In Study 2, we randomized the presentation of these conditions within the neutral and negative blocks (see Supplementary Fig. 1). Each of these sub-blocks was followed by a 30-s resting period.

### Learning and test/feedback

After this procedure, participants learned the 72 object–scene pairs used in the TNT task, plus the six training pairs. Participants were initially told that they participated in an experiment about attention. They were told that this learning procedure was crucial for the next phase (the TNT task) in which we would test their attention. They first learned all the object–scene pairs through a test/feedback cycle procedure. After studying all the pairs for 6 s each, participants underwent test trials in which they were shown the object cue for a given pair for up to 4 s and asked whether they could recall and fully visualize the paired scene. If they responded positively, three scenes then appeared (one correct and two foils took from other pairs of the same emotional valence), and participants were given up to 4 s to decide which scene went with the object cue. After selecting a scene (or if the response window expired), a screen appeared for 1 s indicating whether the recognition judgment was correct or incorrect. In all cases (even if participants indicated that they could fully visualize the associated scene in the first step), each trial ended with the correct pairing appearing onscreen for 2.5 s. Participants were asked to use this feedback to increase their knowledge of the pair. Once all the pairs had been tested, the percentage of total recall was displayed on the screen. If this percentage was below 95% in at least one emotional condition, all the pairs were presented again in a randomized order. This procedure was repeated until the score was above 95%, thus ensuring the correct encoding of the pairs and comparable exposure to all the items. This overtraining procedure was intended to ensure that images would intrude when the cue was presented during the TNT task. Emotional items have also been shown to benefit from stronger encoding in episodic memory^71^. This procedure also helped to further reduce any encoding advantage the negatively valenced scenes might have.

### Criterion test

After learning and practising with the TNT task, participants performed an attentional task for 20 min using an entirely independent image set and intended to address a different question (data not reported here). At this point, participants viewed a brief reminder of all the studied pairs (3 s each), during which they were once again asked to reinforce their knowledge of the pairings. Following this pair refresher, they underwent a final criterion test in which they had to recall the correct image in a similar way to the test/feedback procedure, but this time without any feedback and only once. This allowed us to identify items forgotten before the TNT task and exclude them from the analysis.

### Think/No-Think task

Participants then performed the TNT task, which was divided into eight TNT blocks, each 5 min in length. After two consecutive blocks, a message was displayed on a black screen telling the participants that they could rest. Participants indicated when they wanted to resume the experiment, by pressing any button on the response box. Each block consisted in the presentation of the 24 Think and 24 No-Think items, yielding, across the eight blocks, 256 trials for in the neutral condition, and 256 in the negative condition (disgust and sadness in Study 1, and disgust only in Study 2). Cues appeared for 3 s, framed in either green or red, and centered against a gray background. In Think trials, the cue was framed in green, and participants were told to generate an image of the associated scene as detailed and complete as possible. In No-Think trials, the cue was framed in red, and participants were told that it was imperative that they prevent the scene from coming into their mind, and they should fixate and concentrate on the object-cue without looking away. During the red-cued trials, participants were asked to block out thoughts of the scene by blanking their mind (direct suppression instruction), rather than by replacing the scene with other thoughts or mental images^72^. If the object image came to mind anyway, they were asked to push it out of their mind.

In Study 1, after the offset of each of the Think or No-Think trial cues, participants indicated whether the associated scene had entered their awareness, pressing the button under their right index if it had, and the button under their right middle finger if it had not. As participants only had up to 2 s to do so, they were instructed and trained to do this quickly, without thinking about the associated picture. Their response was followed by a jittered fixation cross randomly lasting 2,400–3,600 ms.

In Study 2, EEG activity was recorded inside a Faraday cage during this procedure. Stimulus presentation was controlled by E-prime Pro on a 17" LCD screen with a 1,280 × 1,024 resolution. To avoid exploratory eye movement, each picture was displayed inside a 400 × 400 pixel square. Participants were seated comfortably in a dimly lit room throughout the experiment, at a distance of 90–100 cm from the screen, and were instructed to minimize blinking and movement during the recording.

Participants had to give feedback concerning the intrusion rate via a response box using their right hand. The fixation cross stayed on the screen until they had done so. In Study 1, participants simply reported either the presence or the absence of intrusive images entering their awareness. In Study 2, they reported the extent to which the associated scene had entered their awareness on a 3-point scale (*Never*, *Briefly*, *Often*), instead of the binary yes/no choice in Study 1. Although we used participants’ responses to classify each No-Think cue as either an intrusion (i.e. *briefly* or *often* response) or a nonintrusion (*never* response) in a binary fashion, this 3-point scale was used to increase participants’ awareness of intrusive memories and ensured that they engaged appropriate inhibitory resources throughout the task to prevent the memory coming to mind, be it only fleetingly or more strongly. In addition, although participants were not given any explicit time constraint on providing their feedback during this procedure, we asked them to make these reports as quickly and intuitively as possible. We did this to prevent them from dwelling too much on the intrusion and potentially recalling the memories.

### Recall

After this procedure, we examined the after-effects of memory suppression via a cued-recall task on all the object–scene pairs, including the 24 baseline scenes (12 neutral and 12 negatives) omitted from the TNT phase. In each cued-recall trial, the cue object was displayed in the center of the screen for 5 s, and participants were told to visually recall the associated picture. If they could recall the associated scene, they were told to press the button under their right index finger, and they then had 15 s to verbally describe the scene in as much detail as possible. Their descriptions were recorded. If they could not recall the associated scene, they had to press the button under their right middle finger, and the next object then appeared on the screen. Participants’ descriptions were checked to ensure that they were consistent with the actual scenes. On average, participants wrongly recalled 0.3% of the scenes in Study 1, and 0.8% in Study 2.

### Post-TNT emotion evaluation

Following the recall task, participants were asked to rate the 72 pictures using the same procedure as before, to measure whether retrieval suppression influenced subsequent affective responses to the scenes. During this task, all the scenes (Think, No-Think and baseline) were presented again, and participants were asked to rate their perceived emotional valence and arousal while their physiological signals were recorded. This procedure enabled us to determine whether retrieval or suppression changed the valence, arousal or cardiac response associated with the item, relative to baseline.

### EEG recording (study 2)

As the EEG cap could be fitted very rapidly (about 15 min), there was only a very short interval between the learning phase and the TNT task. EEG activity was continuously recorded by a GES 300 amplifier (Electrical Geodesics, Inc.), using a HydroCel Geodesic Sensor Net (HGSN-128; Electrical Geodesics, Inc., Eugene, OR, United States) with a dense array of 128 Ag/AgCl sensors^73^. Impedances were kept under 100 k ${\Omega }$^74^, and the EEG channel was referenced to a vertex reference Cz, and the ground to CPPZ (fixed by the EGI system). The signal was sampled at a 20-kHz frequency with a 24-bit A/D and was online (hardware) amplified and low-pass filtered at 4 kHz. The signal was then filtered by a Butterworth low-pass finite impulse response (FIR) filter at 500 Hz and subsampled at 1 kHz. Electro-oculograms were recorded using four electrodes placed vertically and horizontally around the eyes. EEG data were processed offline using Netstation 4.4.2 (Electrical Geodesics Inc., Eugene, OR, USA). The signal was filtered using a 1-Hz Kaiser FIR first-order high-pass filter (which ensures a linear phase and no distortion in the bandwidth), in order to discard DC and very slow waves.

### EEG preprocessing (study 2)

EEG analyses were performed using Version 0.18.1 of the MNE library^75,76^ implemented in Python 3.6, following recent recommendations^77–79^. The EEG pipeline relies on automated steps, in order to promote the sharing of results and subsequent replication. We removed 13 peripheral electrodes (F11, FT11, T9, TP11, P09, I1, Iz, I2, P010, TP12, T10, FT12, F12) from the 115 EEG electrodes, owing to poor impedance quality or lack of contact with the scalp. We low-pass filtered the raw data at a 30-Hz cutoff frequency using a FIR filter and referenced them to a common average reference. Poor sensors were corrected using the RANSAC algorithm implemented in the Autoreject library^80^. Cue onset was adjusted for the delay (15 ms) between trigger generation and the display of the image on the screen in the Faraday cage, measured using photodiodes during preliminary tests. We then segmented the trials from -1,500 ms to 4,000 ms around the cue onset, and applied ICA correction to reduce eye blinks and muscular artefacts, based on visual inspection of the ICA components. Finally, the remaining artefacts were detected and corrected using Version 0.1 of the Autoreject library^80^ with the toolbox’s default parameters.

### Time–frequency analysis (Study 2)

Our analysis focused on the contrasts between the No-Think and Think conditions during the TNT task, and between successful suppression (i.e., nonintrusion) and intrusive trials in the No-Think condition. For the Think condition, we only selected trials where participants reported successful memory reactivation (feedback = 1). We kept all available No-Think trials and extracted the time–frequency representation for each epoch, using the multitaper method between 3 and 30 Hz (27 bins). After this procedure, we epoched the epochs from -500 to 3,000 ms around the onset of the reminder, to avoid edge artefacts, and baseline-corrected them using the percentage method, based on the 500 ms preceding the cue onset. The resulting time–frequency data were then down-sampled to 20-ms time bins for further analysis.

### Statistical analysis

Analyses were performed using custom Python scripts (Python 3.6) together with specific modules for EEG analyses^75^, the Pingouin statistical module^81^, and R packages (R Version 3.4) for factorial analyses^82^ and effect size calculation^83^. Figures, scripts, statistical analyses and part of the data reported in this paper are available at https://osf.io/pjv6d/.

At the behavioral level, items that were not correctly recalled during the criterion test before the TNT phase were systematically excluded from later analyses (Study 1: 1.2%; Study 2: 1.1%). We analyzed behavioral results with an analysis of variance (ANOVA) incorporating all the available factors, and tested the suppression-related effects (e.g. Baseline vs No-Think) with planned comparisons. We performed two-sided comparisons when no a priori hypothesis was formulated, and one-tailed comparisons when we expected to observe specific effects. To improve the clarity of our presentation, the figures do not report all the possible comparisons–only the relevant effects.

For the ECG recordings, we compared neutral and emotional heart rate curves, running a paired *t* test at each time point. We used the fdr_correction() function implemented in MNE Python^75^ to correct for multiple comparisons. For each group, we set the significance threshold at 0.05 to assess the effect of emotions in the first session, and used a one-tailed *t* test to measure the effect of suppression in Session 2.

For the EEG recordings in Study 2, we tested for statistical difference using nonparametric clustering statistics^84^ as implemented in MNE-Python, specifying the connectivity matrix to account for the distance between the electrode sites. We used a one-tailed *t* test and a threshold value of 2.5.

## Results of study 1

### Behavioral results

#### Intrusions

**Figure 2:**
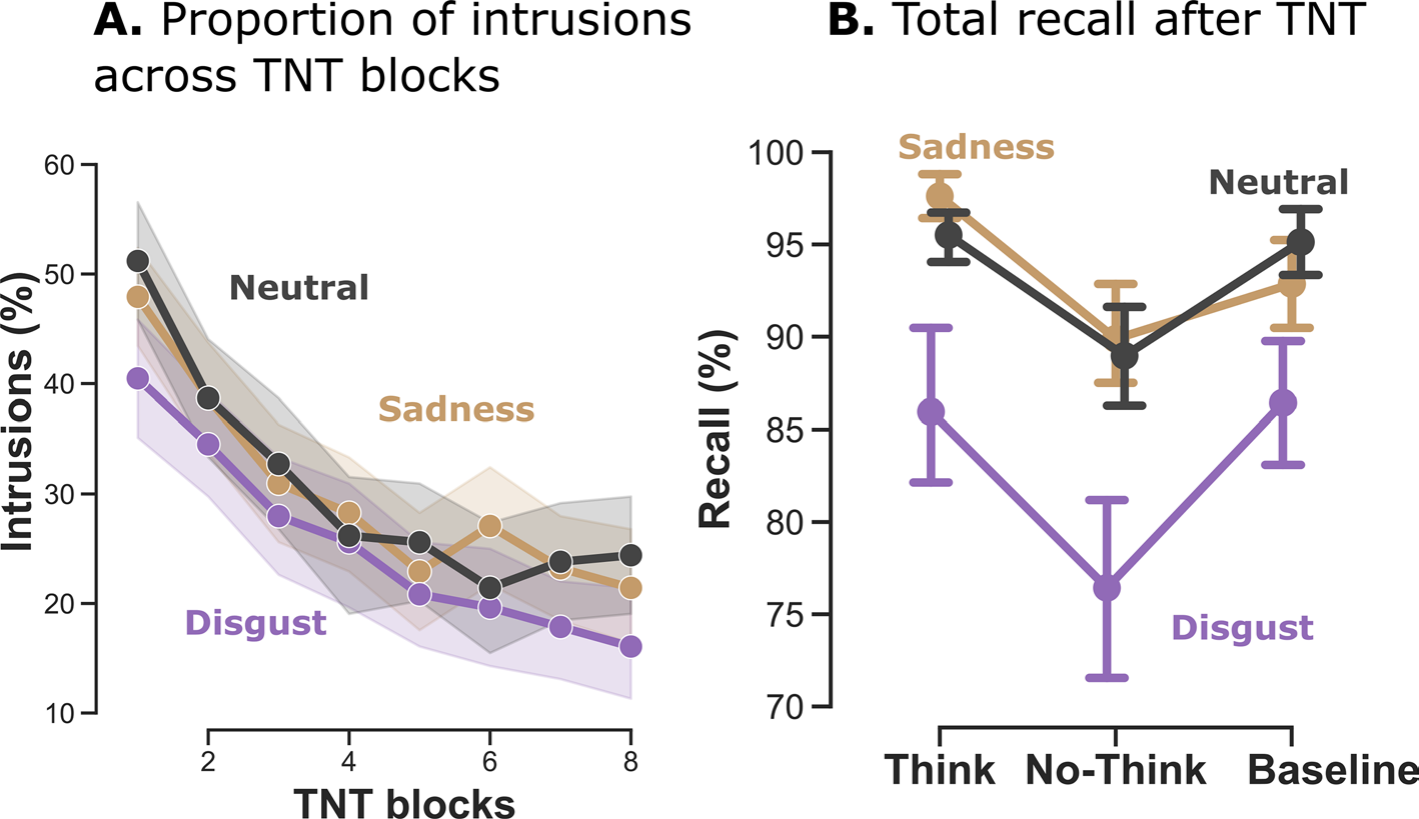
Behavioral indices of inhibitory control over memory in Study 1 (N = 28). **A** Intrusion proportions for No-Think trials (i.e., proportion of trials for which the associated memory entered awareness even though participants had been instructed to inhibit recall) over the eight TNT blocks. Participants increased their ability to control intrusions across the eight TNT blocks. On average, disgusting pictures were reported to be less intrusive than sad or neutral ones.** B** Total recall after the TNT task. Images in the suppression condition (No-Think) were forgotten more than baseline items for both disgusting and neutral emotions. This effect was not significant for sad stimuli.

*Memory intrusions* referred to the involuntary images that popped into participants’ minds in response to a No-Think cue, despite efforts to suppress them. Intrusion ratings were used to isolate reminders associated with intrusive memories and to quantify their occurrence in a binary fashion. For each repetition of a No-Think trial (eight in total), we averaged these binary intrusion reports across all items to compute the temporal dynamics of intrusion proportion. When successful, the inhibitory control of memory recall was characterized by a gradual decrease in the proportion of intrusions during the TNT task^85^. Here, we compared the frequency of intrusions for disgust, sadness and neutral items across the eight TNT blocks for the No-Think condition (Fig. 2A). An Emotion x TNT block ANOVA revealed effects of both emotion, *F*_(1.60, 43.18)_ = 3.82, ${\upeta }_{g}^{2}$ = 0.007, *p* = 0.04, and TNT block, *F*_(3.21, 86.62)_ = 21.00, ${\upeta }_{g}^{2}$ = 0.08, *p* < 0.001, but no interaction between the two, *F*_(7.41, 200.10)_ = 0.78, ${\upeta }_{g}^{2}$ = 0 0.003, *p* = 0.62. Planned comparisons revealed that on average, fewer intrusions were associated with disgusting images than with either neutral, *t*_(27)_ = 2.45, *p* = 0.02, *d* = 0.46, two-tailed, or sad, *t*_(27)_ = 2.04, *p* = 0.05, *d* = 0.38, two-tailed, stimuli.

#### Forgetting induced by inhibition of recall

Preventing memories from entering awareness during the TNT task was associated with a higher level of forgetting of the suppressed scenes during the recall phase, compared with baseline items^22^. We sought to replicate this standard finding by performing an Emotion × Condition (Think vs. No-Think vs. baseline) ANOVA on recall performances (percentage of correctly recalled scenes; see Method). This analysis (Fig. 2B) revealed significant effects of both emotion, *F*_(1.32, 35.51)_ = 16,01, ${\upeta }_{g}^{2}$ = 0.09, *p* < 0.001, and condition, *F*_(1.74, 47.04)_ = 8.17, $\eta_{g}^{2}$ = 0.05, *p* = 0.001, but no interaction between the two, *F*_(2.61, 70.44)_ = 0.86, $\eta_{g}^{2}$ = 0.007, *p* = 0.45. Planned comparisons showed that participants recalled significantly fewer No-Think items than baseline items for disgust, *t*_(27)_ = -2.27, *p* = 0.03, *d* = -0.43, and for neutral, *t*_(27)_ = -3.12, *p* = 0.004, *d* = -0.58, but there was no difference for sadness, *t*_(27)_ = -0.96, *p* = 0.34, *d* = -0.18.

#### Effect of suppression on valence rating

Beyond this well-documented suppression of memory representation, we also wondered whether the ability to control intrusions could be related to changes in the subsequent subjective emotional assessment of the scenes. We examined the SAM valence ratings after the TNT task, normalized using the pre-TNT ratings. An Emotion × Condition ANOVA revealed no significant effects of either emotion, *F*_(1.70, 45.87)_ = 0.28, ${\upeta }_{g}^{2}$ = 0.003, *p* = 0.72, condition, *F*_(1.95, 52.57)_ = 1.17, ${\upeta }_{g}^{2}$ = 0.008, *p* = 0.32, or the interaction between these two factors, *F*_(3.63, 98.10)_ = 0.93, ${\upeta }_{g}^{2}$ = 0.01, *p* = 0.44. Planned comparisons between the No-Think and baseline conditions revealed no significant differences for either disgust, *t*_(27)_ = 0.62, *p* = 0.53, *d* = 0.18, sadness, *t*_(27)_ = 0.75, *p* = 0.45, *d* = 0.14, or neutral, *t*_(27)_ = 0.14, *p* = 0.88, *d* = 0.02. Thus, suppression did not consistently affect the perceived valence of the scenes. We would, however, expect an image’s perceived valence to depend on the context and on the valence of neighboring images, which might modulate decision processes and affective ratings. Moreover, the binary yes/no intrusion scale used in Study 1 (instead of a more subtle 3-point scale) potentially blunted sensitivity to and awareness of intrusions, reducing the engagement of adaptive control processes to purge momentary awareness and inhibition of the perceived valence. These potential issues were addressed in Study 2.

### Physiological results

#### Sadness and disgust decrease heart rate

We then looked at whether the repeated suppression of unwanted memories across blocks of the TNT task was accompanied by persistent modulation of the cardiac response that is normally elicited by the presentation of negative scenes. Before addressing this question, we used the pre-TNT assessment of emotional response to characterize and isolate the specific cardiac autonomic response elicited by negative affect and unpleasant scenes. Here, we analyzed the variations in the number of heartbeats per minute (bpm) triggered by each scene for up to 10 s following image onset. This type of event-related analysis of cardiac response has already been successfully used by researchers to characterize and quantify the heart’s autonomic response to emotion^22^. This method also has the advantage of focusing on the period preceding the valence rating, which more accurately reflects the item’s specific emotional content. It also offers greater flexibility for dealing with spontaneous artefacts, which are problematic in the context of a block analysis. Finally, it reduces the influence of the respiratory signal, whose contamination may be more apparent when cardiac activity is averaged over a longer block duration.

**Figure 3:**
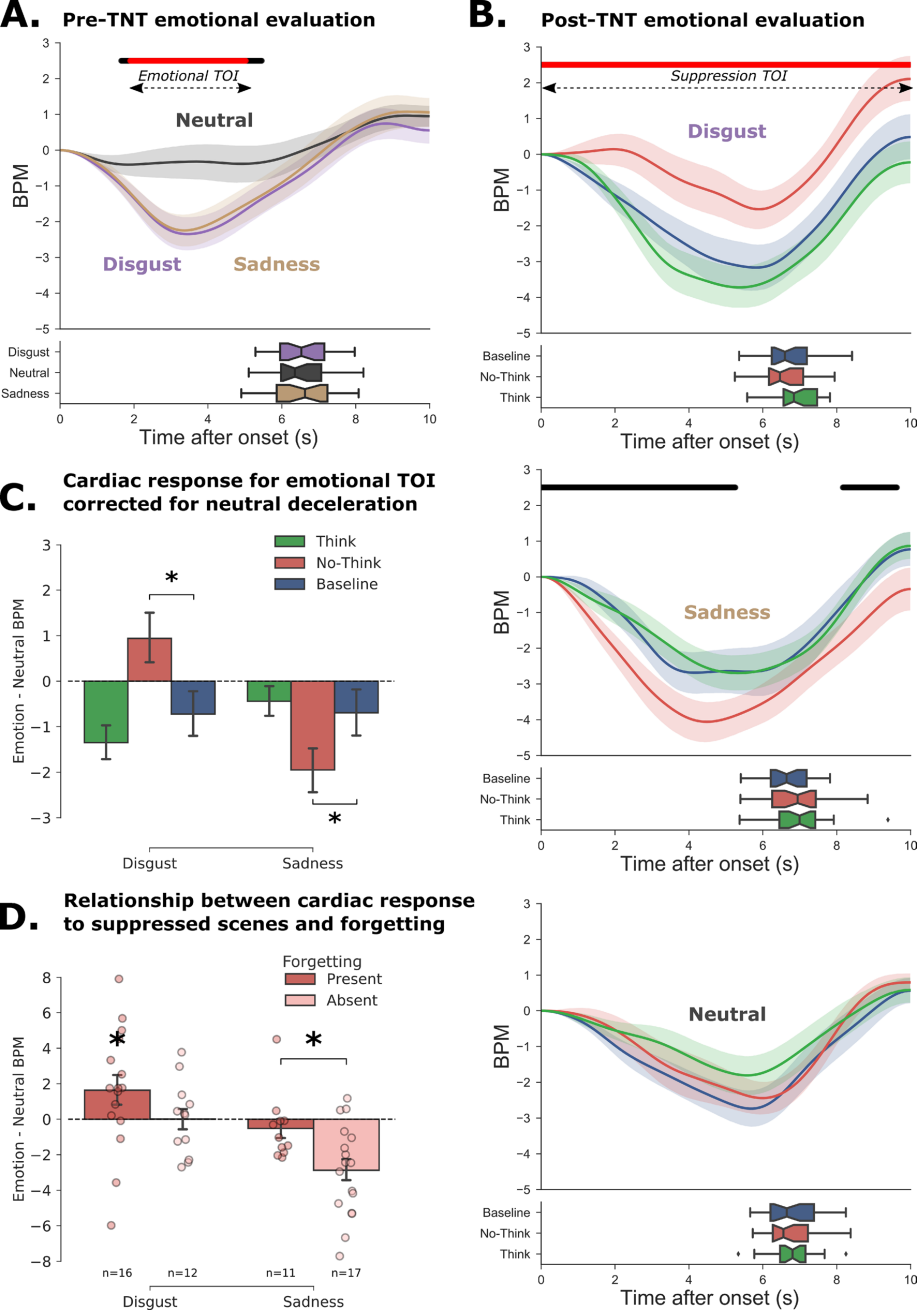
Cardiac response to stimuli before and after the TNT task (Study 1). (**A**) Cardiac response following the initial presentation of the images before the TNT task. The curves represent changes in the number of heartbeats per minute (bpm), taking image onset as the starting point (i.e. 0 sec.). In the lower panel, the boxplots represent the distribution of response times for the valence ratings. (**B**) Cardiac response after the TNT task for disgusting (top panel), sad (middle panel), and neutral (bottom panel) images. For each emotion, the Think (green), No-Think (red) and baseline (blue) items are shown separately. We observed a significantly weaker heart rate deceleration for disgusting No-Think items compared with baseline items, while a stronger deceleration was reported for sad pictures in the suppression condition. In Panels A and B, the black line indicates the significant difference between neutral and emotional (i.e. disgust + sadness) evoked responses, while the red line indicates the remaining significant points after false discovery rate (FDR) correction. (**C**) To control for the stronger deceleration observed for neutral items in the post-TNT evaluation, we computed the difference between the mean bpm for emotional and neutral pictures in the significant emotional time window extracted in the pre-TNT assessment. The reduced or increased deceleration observed for No-Think items was still present for disgusting and sad pictures, even after controlling for neutral deceleration. This suggests that the modulation of the cardiac response following memory suppression could not be attributed to trivial shifts in low-level attention processes. (**D**) To explain the contrasting modulation patterns observed for disgust and sadness, we split participants into two groups according to whether they could suppress emotional scenes from their memory. Participants who were better at suppressing disgusting scenes also showed a weaker heart rate deceleration when they were subsequently exposed to these scenes. By contrast, the stronger heart rate deceleration following the suppression of sad scenes was mostly observed in participants who could not suppress them from their memory.

We compared the averaged bpm curve across the three emotional conditions in the first pre-TNT evaluation (see Fig. 3A). Results showed that scenes categorized as disgusting (maximum deflection peak = -2.34 at 3.48 s, *t*_(27)_ = 4.57, false discovery rate (FDR)-corrected *p* < 0.01, two-tailed paired *t* test) or sad (maximum deflection peak = -2.24 at 3.36 s, *t*_(27)_ = 4.60, *p*-FDR < 0.01, two-tailed paired *t* test) induced a significant deceleration in the heart rate compared with neutral scenes (correction for multiple comparisons across timepoints at *p*-FDR < 0.05). This deceleration in the response to negative scenes occurred within an emotional time of interest (TOI) ranging from 1.89 to 5.03 s.

#### Suppression of unwanted memories inhibits later cardiac deceleration linked to disgusting scenes

We then examined whether repeated cognitive control over emotional memories affected this typical heart rate deceleration we observed in the pre-TNT assessment, by comparing heart rate changes in the post-TNT evaluation following the presentation of Think, No-Think and baseline scenes for the three emotional conditions (see Fig. 3B). We predicted that suppression would reduce the cardiac response that normally characterizes negative scenes. This would be reflected by less deceleration of the cardiac response following the presentation of a previously suppressed unpleasant scene, and thus a positive autonomic response suppression difference compared with baseline (No-Think—baseline timecourses). For neutral scenes, we did not make any specific predictions as to whether retrieval suppression would further dispel residual negativity (which might actually increase bpm rate following the neutral scene), or else simply dampen positive affect for the item, and thus perhaps show a more pronounced deceleration. Given the results observed in the pre-TNT emotional assessment, which showed a greater heart rate deceleration following the presentation of negative scenes within a 1.89–5.03 s time window, we predicted that the effect of memory suppression on cardiac response would arise within a similar time window and would induce a smaller heart rate deceleration. This hypothesis was confirmed for disgusting stimuli, for which we found the expected deceleration compared with baseline, for the whole of the presentation with the peak of maximum difference observed at 4.68 s, *t*_(27)_ = 2.41, *p*-FDR = 0.029, *d* = 0.45. This pattern of results was not observed for sadness, for which No-Think items (-4.05 bpm after 4.48 s) heightened the bpm deceleration compared with Think (-2.69 bpm after 5.33 s) or baseline (-2.68 bpm after 4.16 s) stimuli. The observed difference from baseline did not survive correction for multiple comparisons, *t*_(27)_ = 1.98, *p*-FDR = 0.06, *d* = 0.37, one-tailed. Concerning the neutral condition, a paired *t*-test contrasting Think (-1.8 bpm after 5.57 s) and No-Think (-2.44 bpm after 5.99 s) with baseline (-2.73 bpm after 5.68 s) failed to reveal any significant difference (all *p*s > 0.45, one-tailed).

The cardiac modulation associated with the neutral condition was clearly different in the post-TNT session, compared with the pre-TNT one. We observed a greater deceleration for neutral scenes that was absent in the pre-TNT phase. This discrepancy may have arisen from a significant difference in cardiac frequency between these two measurements. The averaged heart rate over the periods of interest was much higher during the first assessment (80.58 $\pm$ 10.86 bpm) than during the second one (70.80 $\pm$ 9.77, *t*_(27)_ = -5.93, *p* < 10e-5. This difference can be explained by the prolonged seated position during the protocol, as well as by a higher level of stress in the first session, owing to the unusual experimental situation for participants. This reduction of about 10 bpm was enough to increase overall HRV, as this measure is dependent upon the initial heart rate^86^. A higher frequency and lower HRV reduce the time between successive beats, leaving less room for heartbeats to be modulated by attentional and stimulus processing^87^. As a consequence, individuals with higher resting HRV often exhibit greater deceleration when exposed to disgusting stimuli^88^. Here, our results were coherent with this observation, as the averaged deceleration peak across conditions was more pronounced (up to -4 bpm) in the second post-TNT assessment than in the first one.

We then wanted to control for this common deceleration reflecting stimulus processing, in order to isolate the specific emotional response. We subtracted the neutral deceleration (averaged across Think, No-Think and baseline items) from the deceleration waveform of each emotional condition, and then averaged this difference over the emotional TOI identified during the pre-TNT evaluation (see Fig. 3C). Critically, even after controlling for this common neutral deceleration, comparisons between TNT conditions revealed that the memory control condition (No-Think) reduced the subsequent cardiac deceleration in the targeted time window compared with baseline, *t*_(27)_ = 2.65, *p* = 0.01, *d* = 0.50. Furthermore, it induced a stronger deceleration for sad stimuli that was also significantly lower than baseline for this emotion, *t*_(27)_ = -2.21, *p* = 0.03, *d* = 0.41. This pattern reflected a clear effect of the interaction between memory suppression and emotional category on the subsequent modulation of cardiac activity, *t*_(27)_ = 4.25, *p* < 0.001, *d* = 0.8.

Thus, while memory suppression had a greater effect on disgusting scenes and neutralized their impact on the cardiac response (i.e., less deceleration), it paradoxically increased the cardiac deceleration observed for sad scenes. Previous research had reported that the capacity to regulate memory content and affective response are closely related. Behavioral performances reported in Fig. 2, however, suggest that sad images, compared with disgusting ones, were both more difficult to control (higher intrusion rate) and less easily forgotten, despite repeated suppression attempts. We hypothesized that the contrasting suppression effects observed between emotional categories reflected a difference in the ability to control these images. When unsuccessful, inhibitory control may worsen the emotional response associated with the targeted item^27^. From this perspective, the persistence of sad pictures even after repeated attempts to suppress them is presumably accompanied by a stronger emotional response, while the successful control of disgusting scenes yields a parallel inhibition of both memory and cardiac activity.

To test this hypothesis, we divided the participants into two groups for each emotion, according to whether or not they had forgotten the No-Think items following the TNT task. The first group contained participants who had forgotten at least one of the six items (forgetting present group; *n* disgust = 16, *n* sadness = 11), while the second group included participants who had correctly recalled all the items in the No-Think condition (forgetting absent; *n* disgust = 12, *n* sadness = 17) (see Fig. 3D). The ideal procedure would have required us to correct the number of forgotten items with baseline recall. However, this computation led to a small group of high suppressors in the sadness condition (*n* = 7) that was inappropriate for further analysis. Concerning disgust, on average, the forgetting present group exhibited less cardiac deceleration for suppressed items than for neutral ones, *t*_(15)_ = 1.89, *p* = 0.03, *d* = 0.47, one-tailed. This difference between the cardiac responses to disgusting versus neutral No-Think items was not found for the forgetting absent group, *t*_(11)_ = 0.02, *p* = 0.49, *d* = 0.006, one-tailed. Concerning sad pictures, the greater deceleration observed for suppressed scenes was solely observed in the forgetting absent group, and no difference was reported for the forgetting present group, *t*_(25.494)_ = -2.78, *p* = 0.01, *d* = 1.04. This pattern of findings critically suggests that the physiological regulation of emotional response by inhibitory control was influenced by the effective suppression of memory traces. When a memory could not be effectively suppressed, as we observed for sad pictures, the repeated attempts at controlling this unwanted memory and excluding it from awareness actually seemed to have a pernicious effect, accentuating the cardiac deceleration in response to the suppressed material.

### Results of Study 2

Study 1 showed that the cardiac response to unpleasant images can be influenced by inhibitory control exerted over emotional memories encoding these images. The cardiac emotional response was diminished for disgust following suppression, but increased for sadness. We found that this difference may be due to greater memory control abilities over disgust compared with sadness. However, the experimental design of Study 1 included confounding factors that we sought to control in Study 2. Study 1 used a block design for the presentation of the emotional stimuli (see Supplementary Fig. 1). A block presentation has the disadvantage of capturing slow arousal and attentional variations, even with event-related analysis. With this design, participants are also aware of the temporal ordering of the scenes they see. It can be argued that a No-Think block elicits more sustained attention, as participants may gradually increase the attention they pay to forgotten scenes. To control for this bias as much as possible, we conducted a second study featuring a semi-randomized design (see Supplementary Fig. 1). We maintained the separation of emotional categories in time, but randomized the presentation of Think, No-Think, and Baseline stimuli within each block. This meant that participants were aware of the emotional valence of the forthcoming stimulus, but not its condition.

Moreover, to further show that the observed effect was not linked to attentional confound occurring during the emotional assessment, it was crucial to demonstrate that the post-TNT cardiac modulation emerged as a consequence of the neural mechanism engaged during the inhibitory control (i.e., TNT phase). One finding in Study 1 with important implications was the observation of substantial individual differences in the affective consequences of retrieval suppression. This observation, along with similar prior findings^27^, highlighted the existence of interindividual differences in the use of suppression as an effective regulation and coping strategy. It was crucial to clarify whether the large interindividual differences observed in the inhibition of the cardiac response following memory suppression related to differences in the neural mechanisms supporting inhibitory control. We therefore adapted the TNT task utilized in Study 1 to an electroencephalography (EEG) protocol. The combination of EEG and the TNT task has been used in numerous studies, especially ones focusing on the oscillatory dynamics underlying memory suppression^37,38,89^. As EEG requires a large number of trials per condition, we limited Study 2 to disgusting pictures, and did not include sadness. This choice was further motivated by the difficulty of controlling and suppressing sad stimuli, as reflected by recall performances and the proportion of intrusions during the TNT task. Twenty-four new participants underwent this procedure. The full experimental protocol is detailed in the Methods and Materials section. It should be noted that intrusions in Study 2 (see Supplementary Information) were rated on a 3-point scale, in order to increase participants’ awareness of intrusive memories and ensure that they engaged appropriate inhibitory resources throughout the task to limit their awareness.

### Physiological results

#### Study 2 confirmed the influence of memory suppression on cardiac response

**Figure 4:**
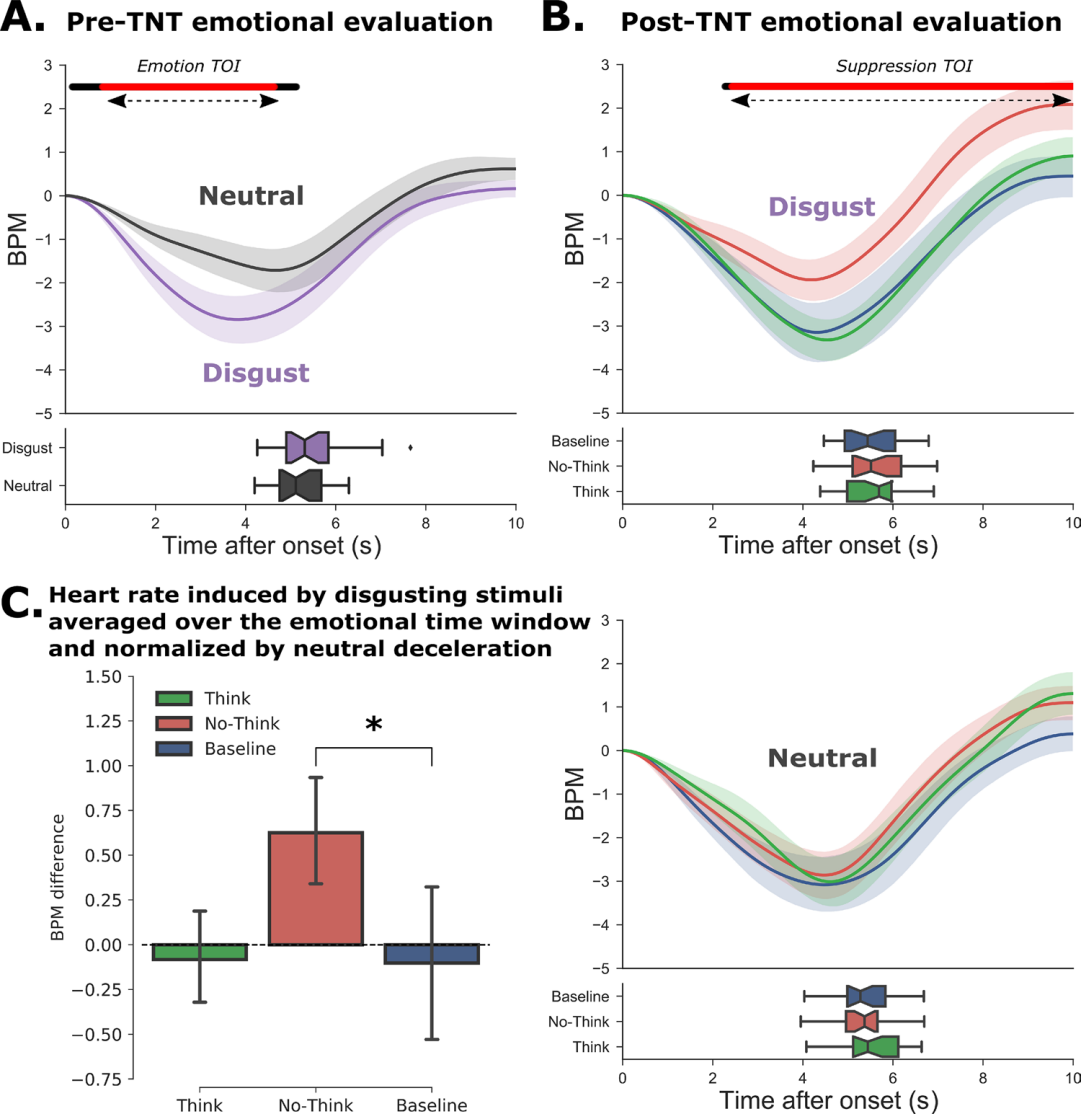
Cardiac response to stimuli before and after the TNT task (Study 2). (**A**) Cardiac response following the initial presentation of the images before the TNT task. The curves represent the change in the number of heartbeats per minute (bpm), taking image onset as the starting point (i.e. 0 sec.). In the lower panel, the boxplots represent the distribution of response times for the valence ratings. (**B**) Cardiac response after the TNT task for disgusting (top panel) and neutral (bottom panel) images. For each emotion, the Think (green), No-Think (red) and baseline (blue) items are shown separately. We observed a significantly weaker heart rate deceleration for disgusting No-Think items, compared with baseline items. In Panels A and B, the black line shows the significant difference between neutral and disgust evoked responses, while the red line indicates the remaining significance after false discovery rate (FDR) correction. (**C**) The inhibition of cardiac response following memory suppression was observed even after controlling for the stronger deceleration observed for neutral items in the post-TNT assessment.

The behavioral findings of Study 1 were largely replicated in Study 2. Participants reported fewer intrusions with disgusting material as compared to the neutral one and we observed a memory suppression effect (No-Think < Baseline) with emotional items during the final recall, but not with neutral items (see Supplementary Information for details). The only exception was the presence of a significant suppression effect on valence ratings collected post-TNT (see Supplementary Fig. 3), suggesting that the block presentation of Study 1 may have compromised the detection of this behavioral effect. As in Study 1, results showed that scenes categorized as disgusting induced a significantly greater heart rate deceleration, compared with neutral pictures in a window of significance (0.81–4.64 s), with the peak of maximum difference observed at 3.28 s, *t*_(23)_ = 3.52, *p*-FDR = 0.009, *d* = 0.71, two-tailed paired *t*-test (see Fig. 4A).

We then compared the heart rate changes in the post-TNT assessment following the presentation of Think, No-Think and Baseline scenes for the items categorized as disgusting (see Fig. 4B). When we compared No-Think and Baseline conditions at each timepoint using FDR correction for multiple comparisons, we found a window of significance (2.41 s to picture offset), with the peak of maximum difference observed at 7.80 s, *t*_(23)_ = 2.45, *p*-FDR = 0.03, *d* = 0.50. No differences were found between conditions for neutral scenes (all *p*s > 0.2).

As in Study 1, we observed a larger overall deceleration in the cardiac response to neutral images in the post-TNT assessment, compared with the pre-TNT assessment. Again, this may have reflected an effect of attentional orientation on cardiac response, which was more likely to occur, given the lower cardiac frequency of participants during the final post-TNT assessment (bpm = 68.64 $\pm$ 10.16), compared with the initial one (bpm = 78.40 $\pm$ 8.72). Critically, the impact of memory suppression on cardiac deceleration still differed significantly from baseline, even after controlling for neutral deceleration in the emotional TOI, *t*_(23)_ = 2.24, *p* = 0.03, *d* = 0.45 (see Fig. 4C), replicating the findings of Study 1.

#### Neurophysiological markers of memory control were correlated with subsequent cardiac modulation

Finally, we wanted to test whether the cardiac modulation we observed was linked to neurophysiological markers of the voluntary control of memory replay. We then recorded the EEG signal of participants in Study 2 while they performed the TNT task. Our goal here was to quantify the interindividual differences in controlling and suppressing the targeted memory during the procedure. We predicted that the electrophysiological features associated with the control and suppression of memory replay would also be related to subsequent cardiac inhibition.

We focused our analysis on two documented electrophysiological signatures of memory control: a reduction in low frequencies associated with suppressed trials compared with memory recall; and an increase in beta frequency associated with successful proactive control during No-Think trials (i.e., nonintrusion vs. intrusion). Our goal here was to compare these electrophysiological markers among participants exhibiting high or low cardiac inhibition during the post-TNT assessment.

**Figure 5:**
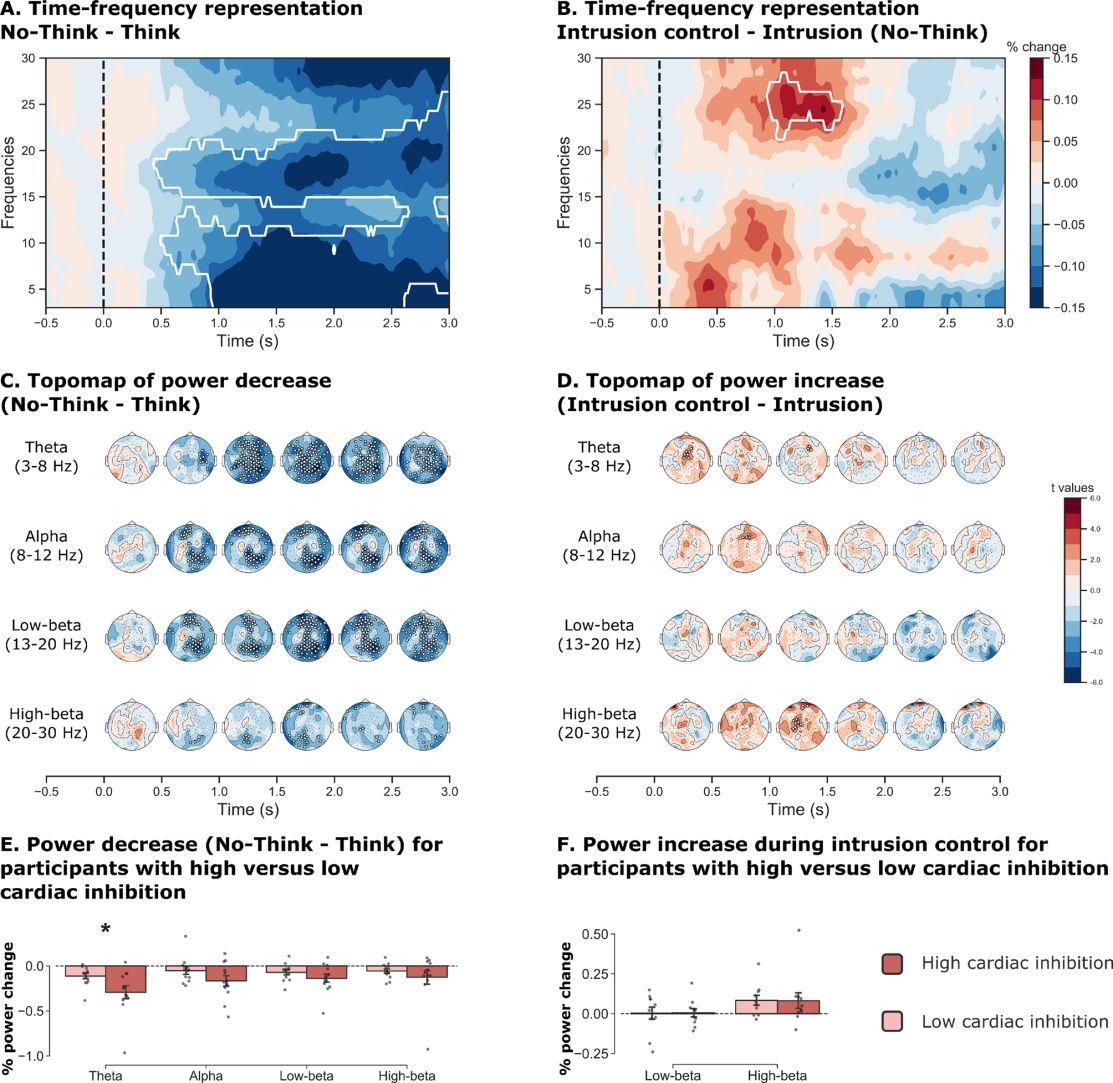
Event-related spectral perturbations associated with memory suppression and their relation to subsequent cardiac inhibition. (**A**) Decrease in frequency power (averaged across the 102 electrodes) during No-Think versus Think trials. This effect was more pronounced for theta (3–8 Hz), alpha (–-12 Hz) and low-beta (13–20 Hz) frequency bands, and appeared around 500 ms after cue onset. White lines indicate a significant cluster (*p* < 0.05). (**B**) Among the No-Think trials, we found an increase in the high-beta frequency band (20–30 Hz) during successful memory control, compared with trials with reported memory intrusions. (**C**) Topographic representation of the event-related perturbation for the No-Think—Think contrast. (**D**) Topographic representation of the event-related perturbation for the nonintrusion—intrusion contrast. The signal change was averaged over 500 ms. Electrodes from significant clusters are shown in white. (**E**) Participants with a higher level of cardiac inhibition also had a greater decrease in the theta frequency band than participants with a lower level of cardiac inhibition. Cardiac inhibition was measured as the averaged heart rate difference between No-Think and baseline items divided by the intersection between the emotional and suppression time windows extracted from the pre- and post-TNT assessments. For each participant, we selected the difference in percentage change between 1,000 and 2,500 ms after cue onset. This difference was not found for the other frequency bands (two-tailed Mann–Whitney rank test). (**F**) We did not find any difference in beta frequency power between participants with high versus low levels of cardiac inhibition (time window of interest between 1,000 and 1,500 ms after reminder onset).

First, for each participant, we averaged the time–frequency representations across the 102 electrode sites and trials, and compared the No-Think and Think conditions (see Fig. 5A). This procedure revealed one significant cluster that passed the permutation test, reflecting a general decrease in the theta, alpha and low-beta frequencies (*p* < 0.001). This decrease lasted from around 500 ms after stimulus onset to the end of the trials. We complemented this general approach with a separate topographic representation of the decrease for each frequency band of interest (theta, alpha, low-beta, and high-beta) (see Fig. 5C). We averaged the power decrease across the frequency bands and six 500-ms time intervals between 0 and 3,000 ms after reminder onset. This procedure confirmed the general decrease observed in Study 1, and highlighted the presence of significant clusters of electrodes that were broadly distributed for the theta frequency band, and restricted more to occipital and frontal areas for the alpha and low-beta bands. These findings replicated sets of previous observations^37,38^. We then looked at whether the suppression of frequency power during No-Think trials (compared with the Think condition) also differed between participants with high versus low cardiac inhibition during the post-TNT evaluation. For each frequency band, we averaged the decrease in power over a 1,000–2,500 ms time window of interest (associated with the strongest difference in the time–frequency representation of Fig. 5A), and compared groups of participants with high versus low cardiac inhibition performances. Participants were divided into two groups, based on the median cardiac inhibition scores (i.e., No-Think minus Baseline), and comparisons were performed using a Mann–Whitney *U* test. We found that participants who exhibited greater cardiac inhibition during the post-TNT test also exhibited a greater reduction in theta frequency power during this period compared with participants with a lower level of cardiac inhibition (*U* = 110, *p* = 0.03, common language effect size (CLES) = 0.76; see Fig. 5E). We did not find any significant differences for either alpha (*U* = 95, *p* = 0.19, CLES = 0.66), low-beta (*U* = 89, *p* = 0.34, CLES = 0.61) or high-beta (*U* = 77, *p* = 0.79, CLES = 0.53) frequencies.

Second, we examined the event-related spectral perturbation associated with the successful control of intrusive memories, compared with the trials labelled as intrusive by participants (see Fig. 5B). We excluded participants with fewer than five trials in one of these conditions from the analysis, as it produced an unreliable mean. This left a total of 20 participants. This procedure revealed six clusters, one of which passed the permutation test, revealing an increase in high-beta frequencies (*p* = 0.027) 1,000–1,500 ms after the presentation of the cue during successful control. A topographic representation (see Fig. 5D) revealed a cluster of central electrodes associated with an increase in the high-beta band during successful control. This finding was consistent with a previous report^90^ showing increased beta activity during successful memory control under similar conditions. Again, we tested whether the augmentation of beta power during successful control differentiated between participants with high versus low cardiac inhibition during the post-TNT assessment. This analysis did not reveal any significant difference (see Fig. 5F) for either low-beta (*U* = 58, *p* = 0.57, CLES = 0.58) or high-beta (*U* = 58, *p* = 0.57, CLES = 0.58) frequency bands.

## Discussion

The persistence of intrusive and unwelcomed mental images is a central feature of numerous psychiatric disorders and can be a source of both physiological and psychological distress. The role of memory suppression in restraining their cognitive and physiological manifestations has been largely debated. Yet, no studies to date have investigated the potential impact of memory suppression on the physiological component of the emotional response. In the present two studies, we showed that the successful inhibition of unwanted memories is accompanied by a long-term modulation of the cardiac activity associated with the presentation of the suppressed distressing material. Findings from Study 1 showed significant inhibition of the cardiac response to disgusting stimuli following memory control. By contrast, suppression of memories associated with sadness heightened the heart rate deceleration compared with baseline, suggesting that suppression may worsen the physiological response to this emotion. This discrepancy was explained by greater difficulty controlling sad pictures and suppressing them in memory, thus supporting the relationship between forgetting and heart rate modulation. To confirm that the cardiac inhibition observed for disgusting material was mediated by neural mechanisms initiated during the inhibitory control of memory awareness, we conducted a second study that solely featured disgusting stimuli, in which we recorded the EEG signal in order to identify the oscillatory dynamics supporting the inhibitory control of memories. An additional motivation for limiting Study 2 to disgusting stimuli was to increase the number of trials per condition and the signal-to-noise ratio for EEG analysis. Study 2 was also designed to control for potential design confounds associated with the presentation of stimuli in blocks during the emotional assessment. Replicating the findings of Study 1, we demonstrated significant inhibition of cardiac activity that was specific to emotion and was therefore not observed with neutral items. Furthermore, we found a link between the suppression of the theta frequency band, a prominent neural marker of memory reactivation, and subsequent inhibition of the cardiac response.

### Inhibitory control of distressing memories induces a long-term cardiac modulation

Our findings supported the notion that memory control constitutes a core mechanism of emotion regulation, subserved by a shared system^25,27,47^. They also extend previous research, by showing that memory suppression impacts not only the cognitive representation of emotions, but also possibly their physiological dimension. Recent studies have reported a link between HRV at rest and inhibitory control performances^91^. This relationship is notably observed using the TNT task^92^, thus supporting the notion that autonomic and cognitive controls are underpinned by a common framework. Critically, however, the current study demonstrated that the control of cognitive representations associated with memory processing can subsequently lead to a long-term change in the cardiac reaction to the suppressed items, going further than the general autonomic traits observed at rest. The effect of inhibitory control over autonomic activity also depends on the initial suppression of the memories: showing reduced physiological activity when memory control is successful, as with disgusting stimuli, but an exacerbated physiological response if memory control is impossible, as we observed with sad memories. Our results provide a consistent argument in favor of a top-down influence of higher-level neurocognitive hierarchies on the autonomic nervous system^49^, and have implications for psychiatric disorders.

### Cardiac inhibition is linked to successful suppression of unwanted emotional memories

Results from Study 1 revealed a contrasting pattern, for while cardiac inhibition was found for disgusting items, suppression of sad memories induced a stronger deceleration. Given that we observed a stronger cardiac deceleration in response to negative scenes in the pre-TNT assessment, this post-TNT deceleration points to a stronger emotional reaction following memory suppression. Images associated with sadness were rated as more intrusive than disgusting ones, and were not associated with suppression-induced forgetting following the TNT phase (see Fig. 2). This pattern of findings suggests that direct suppression was ineffectual for these stimuli. This difference cannot be explained by a lower emotional valence, based on the ratings provided by both the NAPS database^67^ and our own participants. Nevertheless, the sad pictures generally depicted more complex scenes featuring social interactions and facial expressions. Sadness is a social emotion that activates a different pattern of brain areas from disgust^93^. This social complexity may increase the processing of sad scenes through the activation of preexisting social schema, which may, in turn, explain the difficulty of suppressing their memory content. In line with this idea, modulation of amygdala activity during directed forgetting attempts is observed for scenes involving disgust reaction, but not those related to sadness^94^. Previous studies have suggested that when unsuccessful, memory suppression actually worsens the distressing symptoms^7^. Suppression can be ineffectual when memories are consolidated^95^ or too strongly reactivated^96^. It can also accentuate the emotional response if individuals have poor control abilities^27^. To test the hypothesis that the greater deceleration observed for sad items was due to ineffectual memory suppression, we compared the cardiac modulation for sad and disgusting images in participants who forgot at least one image in the post-TNT recall versus those who did not forget any (see Fig. 3). Results showed that the amplitude of the cardiac modulation depended on participants’ forgetting rate, suggesting that the worsening of the cardiac response occurred after unsuccessful control of sad memories. Thus, although cardiac modulation is influenced by memory suppression, the direction of this influence depends on participants’ ability to successfully control and suppress distressing images in memory. These findings echo well with previous studies reporting that individuals who show better deployment of control resources experience fewer intrusive memories^23,27,97^, greater suppression of perceptual memory traces^30^, greater modulation of the emotional response^27^, and greater forgetting^35^.

### Better cardiac inhibition is associated with a theta decrease during memory suppression

The current data support a close connection between the successful control of memories and the subsequent inhibition of the cardiac response during the presentation of the suppressed scenes. However, this cardiac inhibition does not necessarily imply that the memory control network has reduced some aspect of memory activation in the service of affect regulation. Cardiac inhibition may instead arise, for instance, from other sources of attentional modulation engaged during the sensory processing of the suppressed scenes, but independent of the neural mechanisms engaged during repeated attempts to suppress the emotional memories. To corroborate the link between inhibitory control and heart rate modulation, we measured the event-related spectral perturbation associated with memory control for participants with a high or low level of cardiac inhibition. In line with previous studies^37,38^, we observed a reduction in low frequency (< 20 Hz) during memory suppression (No-Think trials) compared with voluntary memory recall (Think) (see Fig. 5). Interestingly, we also found that participants exhibiting a higher level of cardiac inhibition during the post-TNT evaluation also showed a greater decrease in the theta frequency band. This corroborates the idea that the cardiac modulation after the TNT task may have been induced by the reduction or suppression of voluntary recall mechanisms during the No-Think trials, compared with the Think trials.

For the No-Think trials, we observed a greater increase in high-beta activity during trials associated with successful control, compared with trials triggering intrusive memories. This result is in line with a previous report^90^, and indicates that fast, transient increases in beta frequency power in prefrontal electrodes may be physiological markers of top-down proactive control^63^. Critically, these patterns have also been found in other tasks engaging inhibitory control, such as action stopping^63^. Here, we failed to find a clear relationship between the averaged amplitude of this increase and subsequent cardiac inhibition. This suggests that cardiac inhibition is not mediated by control mechanisms associated with the beta band. However, both nonintrusive and intrusive reminders require the deployment of control resources, and may generate a high level of physiological inhibition. The additional control demand triggered by intrusive memories and usually associated with hippocampal top-down processes in a connectivity pattern^16,23,27,97^ may not manifest itself in the oscillatory dynamics observed at the scalp level. Moreover, memory intrusions and their reflexive inhibition are both presumed to be brief and transient, as suggest by event-related potential results^98^. Measuring averaged frequency power across conditions is therefore likely to flatten the trial-wise dynamics and give a biased estimation of individual suppression deployment.

This difficulty was less present with the theta frequency band, as we used sustained voluntary recall as a comparison. The decrease in low-frequency power during memory suppression may reflect inhibition of memory recall processing. This is especially the case for the theta frequency band, which is a marker of memory encoding and recall in the medial temporal lobe^39^. However, because the oscillatory dynamics were measured with EEG on participants’ scalp, the contribution of different cortical sources must be interpreted with caution. Although Depue et al.^89^ reported an increase in theta power for no-think trials, our findings, like others^37,38^, pointed instead to the suppression of theta activity during these trials. This discrepancy may reflect an early and brief increase in proactive prefrontal cognitive control, and a more sustained adaptive suppression of theta oscillatory dynamics in the medial temporal lobe during the suppression trials^38^. We assumed that the former effect occurred in the early phase of the stimuli, especially when participants could anticipate an upcoming reminder cue (< 1 s), whereas the latter was observed throughout the later time window of the reminder cue.

### Implications for psychophysiology

HRV is a central manifestation of emotional experience^48,99^. Frequency changes reflect an adaptation to new environmental outcomes that covertly influences perception and decision making^100^. According to the neurovisceral integration model^49^, amygdalar activation, modulated by higher prefrontal areas^51^, plays a key role in this process^101^. This structure can trigger the acceleration or deceleration of the autonomic response via direct connections to the hypothalamus and brainstem circuits, and a complex interplay of connections to the sympathetic and parasympathetic networks^102^. For example, negative experiences, which may induce hyperactivation of the amygdala, are traditionally thought to induce heart rate acceleration. This assumption holds for fear, stress and anxiety, which all require greater sympathetic recruitment to support defensive behaviors. However, the literature indicates a more mixed pattern for disgust and sadness. For example, disgust is more consistently associated with a cardiac deceleration when it relates to mutilation, but an acceleration for contamination-related stimuli (see Kreibig et al.^48^ for a review). This may indicate a need to slow down the organism to avoid blood loss. Then again, sadness can elicit an increase in cardiac activity under high intensity and crying sadness, and a decrease during noncrying or anticipatory sadness^48,58^. These changes are associated with the reduction in sympathetic influence. Critically here, the two emotions we manipulated (mainly mutilation-related disgust and non-crying sadness; see Supplementary Material) induced a clear cardiac deceleration during the pre-TNT assessment, which we interpreted as a marker of emotional response in line with preexisting literature.

Deceleration of cardiac frequency is one of the possible adaptive behaviors to external and internal threats. This reflex can denote either a need to slow down the activity of the organism to avoid blood loss or contamination, a freezing response observed in many species when faced with an extreme and unbearable level of threat, or even the anticipation of upcoming fight or flight behavior. These deceleration patterns can be accompanied by both increased sensory processing to prepare for future behaviors, and motor inhibition to avoid detection by predators. According to polyvagal theory^52^, these reactions are mediated by the parasympathetic influence of the older branch of the vagal nerve. Here, Studies 1 and 2 both highlighted inhibition of this pattern after memory suppression for the disgusting stimuli, possibly reflecting the intervention of a modulatory factor through the influence of the ventral branch of the vagus^52^. We can assume here that this change was a consequence of the down-regulation of the amygdala for the suppressed items observed during the TNT task^27^. This influence is also liable to change the associated autonomic response, and because this absence of deceleration was observed over a small timescale (< 6 s), it is more likely to have been mediated by the parasympathetic network^103^. We therefore suggest that the physiological regulation of emotional experience after the successful suppression of an unwanted memory is probably exerted by the most recent component of the vagal nerve. This opposing mechanism may then partly override the automated slowdown response exerted by the oldest, dorsal part.

Although we found a consistent cardiac modulation in Study 1 and Study 2, the subjective valence reported by the participants was only increased after memory suppression in Study 2. This questions both the association between the physiological and experiential dimension of emotions and the dual influence of memory suppression on these components. The association between the physiological aspects of emotions and subjective valence judgements has large interindividual variability and can be influenced by factors like gender or emotion category^104,105^. In Study 2, we also randomized the presentation of TNT conditions (see Supplementary Fig. 1) to control for a possible block effect. Because we only observed modulation of emotional valence after TNT under this randomized effect, we hypothesized here that the process of valence judgements can be biased by this task structure and explain some of the discrepancies between physiological arousal and the subjective evaluation of valence.

### Implications for psychiatric disorders

Our results constitute promising evidence for the physiological regulation of disgust when memory suppression via inhibitory control is effective. Studies 1 and 2 both contradicted the notion that all negative stimuli are more difficult to control, and were instead in line with previous studies reporting less frequent intrusions^27^ and greater memory suppression^25^ for negative memories. Here, it is important to note that the emotional effect was specific to disgusting stimuli, and appeared more contrasted for sad stimuli. This pattern of behavioral findings may reflect the existence of a greater motivational force driving the need to control and suppress memories for disgusting sights. Then again, it may also reflect the greater natural need to avoid disgusting pictures and gradually exclude disgusting items from memory after encoding^106^. This is compatible with the fact that we observed generally poorer recall of disgusting stimuli, compared with sad or neutral scenes. Although we could not tease these two hypotheses apart in the current study, it is worth mentioning that they were not mutually exclusive and were both rooted in the natural desire to avoid and reject emotional disgust.

The emotion of disgust plays a decisive role in the development and maintenance of numerous psychiatric conditions, including anxiety and obsessive–compulsive disorder^107^. Although this emotion does not always dominate the symptoms or induce the most salient ones, its experience can favor cognitive biases toward particular stimuli or situations, and contributes to the maintenance or increase of more debilitating manifestations such as fear and state anxiety. For example, the experience of disgust is central in OCD^108^. A high propensity to food-related disgust has also been described as a defensive mechanism for avoiding calorie intake in anorexia nervosa^109^. These examples are straightforward, as they refer to standard animal, blood or food-related stimuli, but it is worth pointing out that disgust also encompasses a moral dimension for humans^110^ that we did not explore here. Moreover, while depressive syndromes mostly relate to the experience of sadness and do not involve a high propensity to externally-oriented disgust, self-oriented disgust is regarded as an important dimension of these conditions^111^. Although it is not clear whether more complex and resistant emotional states reflect the disruption of control mechanisms associated with memory suppression, the current findings raise the hope that interventions focused on training memory control could, in principle, reduce intrusions, all the while dampening negative affect and physiological reactions.

By showing that retrieval suppression contributes to cardiac regulation, the present findings may offer insights into the mechanisms underlying intrusive symptoms in psychiatric disorders. In this context, variability in the cardiac response appears to be a relevant marker of both inhibitory control abilities and emotion regulation. Although the regulation of cognitive representations associated with intrusive symptoms may not be appropriate for resistant and complex emotional states involving sadness, it may constitute a promising avenue for dampening other emotional experiences and their physiological roots in the causal chain of psychiatric symptoms. This notion could be clarified through additional researches focusing on the roots of inhibitory control and its alteration in psychiatric disorders, the resistance of some representations to forgetting and the physiological mechanisms mediating autonomic control in the context of emotional memories regulation.

## Supplementary information

Supplementary file1
