# Supplementary material for: Long-term modulation of cardiac activity induced by inhibitory control over emotional memories
Source: Sci Rep. 2020 Sep 14;10:15008. doi: 10.1038/s41598-020-71858-2 (PMC7490349; doi:10.1038/s41598-020-71858-2)
Supplement: Supplementary file 1 — Supplementary file1 [file 41598_2020_71858_MOESM1_ESM.docx]

**Long-term modulation of cardiac activity induced by inhibitory control over emotional memories**

Nicolas Legrand^1^, Olivier Etard^2, 3^, Anaïs Vandevelde^3^, Melissa Pierre^1^, Fausto Viader^1^, Patrice Clochon^1^, Franck Doidy^1^, Denis Peschanski^4^, Francis Eustache^1^, and Pierre Gagnepain^1*^

1 - Normandie Univ, UNICAEN, PSL Research University, EPHE, INSERM, U1077, CHU de Caen, Neuropsychologie et Imagerie de la Mémoire Humaine, Caen, France

2 - CHU de Caen, Service des explorations fonctionnelles du système nerveux, Caen, F-14000, France

3 - Imagerie et Stratégies Thérapeutiques de la Schizophrénie (ISTS), Normandie Univ, UNICAEN, Faculté de médecine, CYCERON, Caen F-14000, France

4 - Université Paris I Panthéon Sorbonne, HESAM Université, EHESS, CNRS, UMR8209, 75231 Paris, France.

* Corresponding author. Email: [pierre.gagnepain@inserm.fr](mailto:pierre.gagnepain@inserm.fr)

Supplementary information

## Experimental design


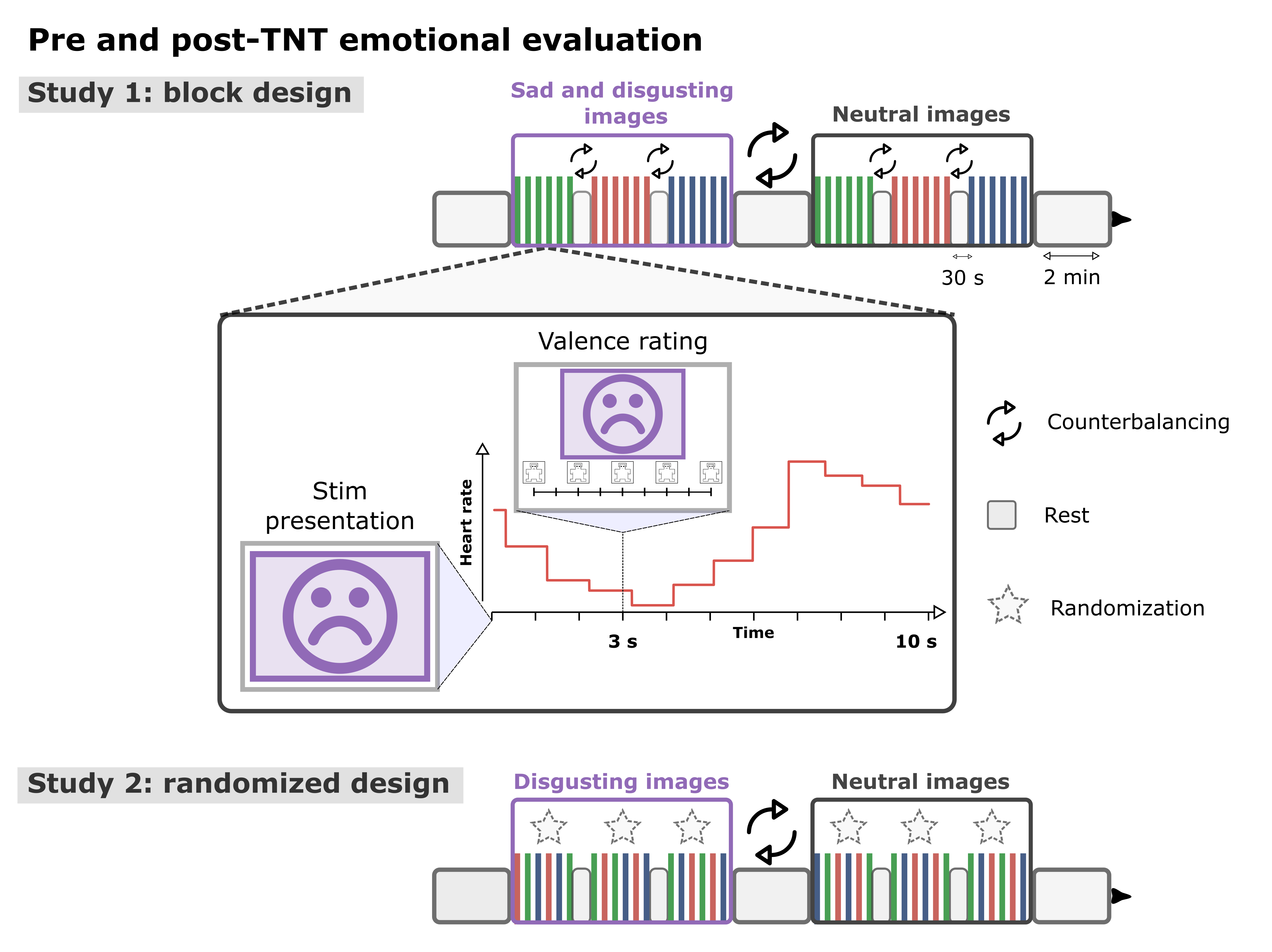


**Supplementary Figure 1.** **Pre- and Post-TNT emotional evaluation.** Emotional assessment in Study 1 included a block design during which stimulus presentation alternate between discrete epochs of scenes drawn from the same condition (i.e.Think, No-Think, and Baseline). This design was intended to optimize the detection of differences in heartbeats which may have only arisen in the low-frequency range. One potential problem with blocked designs, however, is that the response to events within a block may be confounded by the context of their occurrence (e.g. when participants become aware of the blocking and may alter their strategies/attention as a consequence). To control for that potential bias, Study 2 used a design in which the presentation of Think, No-Think, and Baseline items was randomized and unpredictable. However, we kept emotion and neutral conditions presented in separated blocks to control for long-term autonomic change induced by emotions that may spread over and modulate cardiac response to neutral scenes. In addition, in Study 1, an arousal scale was also presented at the bottom of the screen, ranging from 1 (corresponding to a calm face on the far left of the scale) if a picture made them feel completely relaxed, to 9 (corresponding to an excited face on the far right if a picture made them feel completely aroused). If participants felt neutral, neither relaxed nor aroused, they were then instructed to press the square under the figure in the middle position. This last change explains the difference in mean response time between Study 2 and Study 1.

## Behavioural results - Study 1


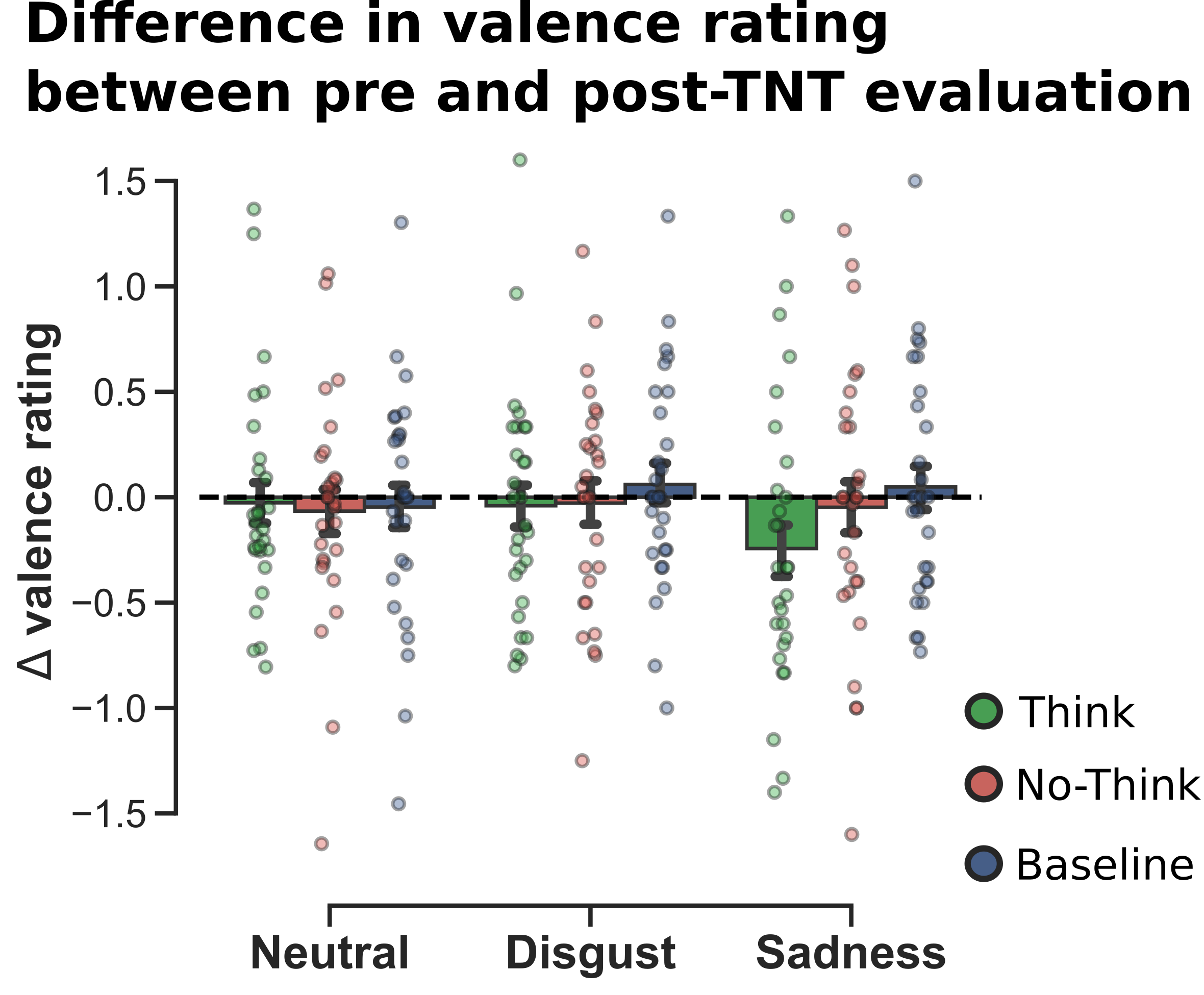


**Supplementary Figure 2. Difference in valence rating between pre- and post- TNT evaluation in Study 1.** We found no significant differences in term of subjective rating for the No-Think pictures as compared to baseline in any of the three emotional conditions. Shaded error bands and error bars represent the bootstrapped standard-error of the mean.

## Behavioural results - Study 2

We applied the same sequence of analyses for the behavioural data in Study 1 and Study 2.

## Intrusions

We tested if the item categorized as disgusting produced less intrusion than neutral scenes as we previously observed. We compared the frequency of intrusion of Disgust and Neutral items over the eight TNT blocks for the No-Think condition (see Supplementary Figure 3 B.). An Emotion x TNT blocks ANOVA showed both an effect of Emotion (F_(1, 23)_ = 19.49, $\eta_{g}^{2}$= 0.01,  *p* < 0.001) and TNT blocks (F_(3.82, 87.88)_= 24.75, $\eta_{g}^{2}$ = 0.08, p < 0.001) but no interaction between these two factors (F_(4.81, 110.61)_ = 0.73, $\eta_{g}^{2}$ = 0 .002, p = 0.60).

## Inhibition of recall induces forgetting

An Emotion × Condition ANOVA on participants’ memories reflected by the Yes/No answer (see Supplementary Figure 3 A.) revealed a significant effect for Emotion (F_(1,23)_= 12,17, $\eta_{g}^{2}$ = 0.07, *p* = 0.002), Condition (F_(1.56,35.89)_ = 16.85, $\eta_{g}^{2}$ = 0.11, *p* < 0.001) but no interaction between those two factors (F_(1.44,33.05)_= 2.52, $\eta_{g}^{2}$ = 0.02, p = 0.11). Comparisons indicated that participants recalled significantly less No-Think item than Baseline for Disgust (t_(23)_= -4.40, *p* = 0.0001, *d* = 0.89, one-tailed), while this effect was not significant with neutral items (t_(23)_ = -1.59, *p* = 0.06, *d* = 0.32, one-tailed). This result parallels previous works ^1,212,72^ suggesting that emotional stimuli can be efficiently controlled and forgotten. These patterns of behavioural findings replicate observations made in Study 1 and suggest that emotional disgust may increase the motivation and the desire to reduce the momentary awareness associated with this unpleasant and unwanted memories. However, similarly to results reported in Study 1, disgusting items were globally more forgotten than neutral ones, which suggests that both a diminished memory of the items and a stronger motivation to control them could have produced the observed patterns.

## Effect of suppression in the subjective evaluation

We then examined the SAM valence rating after the TNT task adjusted by pre-TNT evaluations (see Supplementary Figure 3 C.). An Emotion × Condition ANOVA revealed a significant effect of Emotion (F_(1, 23)_ = 9.78, $\eta_{g}^{2}$ = 0.1, *p* = 0.005) but not for Condition (F_(1.97, 45.38)_ = 1.43, $\eta_{g}^{2}$ = 0.009, *p* = 0.25) or the interaction between those two factors (F_(1.94, 44.63)_ = 1.42, $\eta_{g}^{2}$ = 0.01, *p* = 0.25). Planned comparison showed a significant higher valence rating for No-Think items as compared to Baseline items for Disgust (t_(23)_ = 1.8, *p* = 0.04, *d* = 0.36, one-tailed). A difference was also observed for this emotion between Think, and Baseline images (t_(23)_ = 1.66, *p* = 0.05, *d* =0.33, one-tailed) while no difference was found between Think and No-Think conditions (t_(23)_ = 0.01, *p* = 0.98, *d* < 0.01, two-tailed). Concerning Neutral scenes, we did not found similar increase of the valence rating for No-Think items as compared to baseline (t_(23)_= 0.74, *p* = 0.46, *d* = 0.15). These findings show that suppressing disgusting scenes from memory may also alter the emotional quality of those memories so that their reappearance triggers less negative affect. This result parallels a recent study showing affective devaluation of words and objects after a TNT procedure ^3^. Interestingly changes in affect do not arise for Neutral scenes suggesting that suppressing unpleasant memories may entail additional inhibitory effects not present for Neutral memories. Retrieval during Think trials measurably alters the perceived affect of the scenes to some extent, suggesting the existence of some form of habituation effect following repeated retrieval.

## Behavioural indices of inhibitory control and evolution of emotional appreciation in Study 2 (n=24)


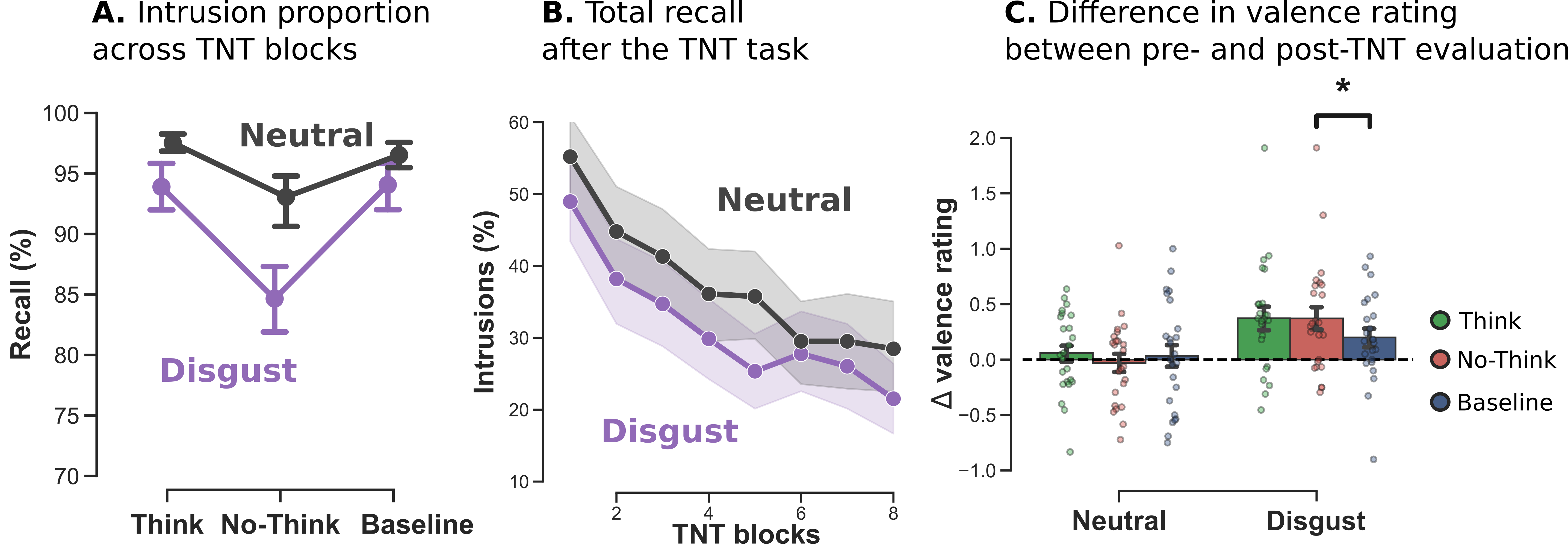


**Supplementary Figure 3.** **A.** Intrusions proportions for No-Think trials (i.e. the proportion of trials where the associated memory entered into awareness while participants were instructed to inhibit recall) over the eight TNT blocks. Participant increased their ability to control intrusion over the eight TNT blocks. On average, disgusting pictures were reported less intrusive than neutral ones. **B.** Total recall after the TNT procedure. Images in the suppression condition (No-Think) were more forgotten than baseline for disgusting items, an effect that was not found for the neutral emotion. **C.** Differences in valence rating between pre- and post- TNT evaluation. We found a significant difference in valence rating between No-Think and Baseline pictures for disgust, but this effect was not significant for neutral items. Shaded error bands and error bars represent the bootstrapped standard-error of the mean.

## Images reference

### Study 1 - NEUTRAL NAPS reference

Animals [_043_h, _088_h, _097_h, _105_h, _108_h, _132_h, _155_v, _213_h], Faces [_060_h, _061_h, _065_h, _123_h, _184_h, _211_h, _219_v, _292_h, _306_v], Objects [_029_h, _049_h, _164_h, _200_h, _242_h, _270_h], People [_026_h, _028_h, _035_h, _091_h, _095_h, _097_h, _100_h, _148_h, _149_h, _151_h, _164_h, _179_h, _249_h].

### Study 1 - DISGUST NAPS reference (mainly mutilation related disgust)

Animals [_039_h, _041_h, _062_h, _065_h], Faces [_156_h], Objects [_006_h, _010_h, _011_h, _022_h, _125_h, _126_h], People [_019_v, _077_v, _090_v, _205_v, _209_v, _220_h, _239_h].

### Study 1 - SADNESS NAPS reference

Animals [_013_h, _053_h, _067_h, _077_h], Faces [_011_h, _012_v, _013_h, _019_h, _034_h, _149_v, _279_h, _294_h], People [_001_h, _002_v, _127_h, _133_h, _143_h, _147_h].

### Study 2 - NEUTRAL NAPS reference

Animals [_043_h, _088_h, _097_h, _105_h, _108_h, _132_h, _155_v, _213_h], Faces [_060_h, _061_h, _065_h, _123_h, _184_h, _211_h, _219_v, _292_h, _306_v], Objects [_029_h, _049_h, _164_h, _200_h, _242_h, _270_h], People [_026_h, _028_h, _035_h, _091_h, _095_h, _097_h, _100_h, _148_h, _149_h, _151_h, _164_h, _179_h, _249_h].

### Study 2 - DISGUST NAPS reference (mainly mutilation related disgust)

Animals [_008_v, _018_h, _039_h, _041_h, _062_h, _065_h, _078_h], Faces [_156_h, _296_h, _368_h], Objects [_006_h, _007_h, _010_h, _011_h, _013_h, _021_v, _022_h, _023_v, _088_h, _106_v, _125_h, _126_h, _154_h], People [_019_v, _077_v, _090_v, _204_v, _205_v, _209_v, _213_v, _217_h, _220_h, _223_h, _230_h, _239_h, _247_v].

# References

1. Depue, B. E., Curran, T. & Banich, M. T. Prefrontal Regions Orchestrate Suppression of Emotional Memories via a Two-Phase Process. *Science* **317**, 215–219 (2007).

2. Lambert, A. J., Good, K. S. & Kirk, I. J. Testing the repression hypothesis: Effects of emotional valence on memory suppression in the think – No think task. *Conscious. Cogn.* **19**, 281–293 (2010).

3. Vito, D. D. & Fenske, M. J. Suppressing memories of words and familiar objects results in their affective devaluation: Evidence from Think/No-think tasks. *Cognition* **162**, 1–11 (2017).
